# Synergistic Effects of Microenvironmental Heterogeneity and Propagule Pressure Drive the Adaptive Expansion of a Wetland Invasive Plant in an Arid Region

**DOI:** 10.1002/ece3.72272

**Published:** 2025-10-26

**Authors:** Shengtianzi Dong, Wenxuan Zhao, Tiantian Qin, Hongyang Chen, Wenchao Guo, Hanyue Wang, Hegan Dong

**Affiliations:** ^1^ College of Life Sciences Shihezi University Shihezi China; ^2^ Xinjiang Production and Construction Corps Key Laboratory of Oasis Town and Mountain‐Basin System Ecology Shihezi China; ^3^ College of Grassland Science Xinjiang Agricultural University Urumqi China; ^4^ Xinjiang Key Laboratory of Agricultural Biosafety Urumqi China

**Keywords:** biological invasion, climatic tolerance, microhabitat refugia, propagule pressure, topographic moisture gradient

## Abstract

Understanding how invasive species establish in environments outside their typical climatic range is crucial for predicting and managing biological invasions. This study experimentally assessed the survival mechanisms of giant ragweed ( *Ambrosia trifida* L.), a wetland plant, in the arid Yili Valley of Northwest China—a region with climatic conditions markedly different from its known distribution. Using comprehensive niche analysis methods (PCA and kernel density estimation), we confirmed that the Yili Valley represents a novel climatic space for this species, with mean annual precipitation (200 mm) far below its typical requirements (> 800 mm). Field experiments demonstrated that topographically driven moisture accumulation creates critical microhabitat refugia, with lower slope positions maintaining significantly higher soil moisture than middle and upper slopes throughout the growing season. Linear mixed‐effects models revealed that slope position (*F* = 109.77, *p* < 0.001) and propagule pressure (*F* = 225.55, *p* < 0.001) were the primary drivers of seedling establishment, with their significant interaction (*F* = 5.18, *p* < 0.001) indicating that high propagule input can partially compensate for suboptimal moisture conditions. Seeds from different collection years (2010–2022) showed variable performance, with the collection year significantly affecting long‐term survival (*F* = 20.24, *p* < 0.001) but not initial establishment, suggesting potential population‐level changes during the invasion period that warrant further investigation with common garden experiments. Our findings demonstrate that microenvironmental heterogeneity provides crucial stepping stones for invasive species in climatically unsuitable regions, highlighting the importance of considering fine‐scale habitat variation in invasion risk assessments.

## Graphical abstract

*Ambrosia trifida* invades arid Yili Valley, a striking climatic niche departure. Synergy of microrefugia and propagule pressure drives arid invasion success. Latent invaders should be managed early to pre‐empt their rapid evolution and impact.

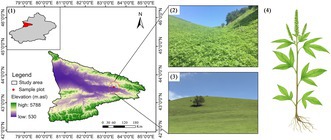

## 1 Introduction

Biological invasions represent a major component of global environmental change, with profound impacts on biodiversity, ecosystem functioning, and human welfare (Shackleton et al. 2018; Linders et al. 2019; Carneiro et al. 2024). A fundamental principle in invasion ecology is that species distributions are primarily constrained by their climatic tolerances (Hutchinson and Vankat 1997; Araújo et al. 2005). Since species' fundamental niches are typically difficult to quantify, Species Distribution Models (SDMs) based on realized climatic niches have become the primary tool for predicting invasion risk (Thuiller et al. 2005; Broennimann et al. 2007; Dallas and Kramer 2022). The theoretical foundation of this approach is the climatic niche conservatism hypothesis, which assumes that species' climatic niches remain conservative across space and time (Soberón and Townsend Peterson 2011). However, the niche conservatism hypothesis has been subject to considerable debate (Losos 2008; Hua and Wiens 2013), and mounting evidence suggests that some invasive species successfully establish in regions with climatic conditions that appear unsuitable based on their native or previously invaded ranges (Banerjee et al. 2019; Christina et al. 2020; Lian et al. 2022).

The successful establishment of species in apparently unsuitable climates challenges our understanding of the factors limiting species distributions and the mechanisms enabling range expansions. While evolutionary adaptation has been proposed as one mechanism (Colautti and Barrett 2013; Oduor et al. 2016), the immediate ecological processes that enable initial survival and population persistence deserve primary attention. Microenvironmental heterogeneity, particularly in topographically complex landscapes, can create localized conditions that differ substantially from regional climate averages (Forester et al. 2016; Chaturvedi and Chatterjee 2025). These microhabitat refugia may serve as crucial stepping stones for species establishment in otherwise unsuitable regions.

In arid and semi‐arid ecosystems, while regional climate patterns largely determine water availability, localized water access can be significantly enhanced through nonclimatic mechanisms (Thuiller et al. 2005; Dallas and Kramer 2022). Topographic variation can create substantial heterogeneity in soil moisture through runoff accumulation, shading effects, and differential evapotranspiration that operate independently of regional precipitation patterns (Leishman and Thomson 2005; Zhang et al. 2019; Fang et al. 2023). For moisture‐dependent invasive species, these topographically driven moisture gradients may provide essential refugia that enable persistence despite unfavorable regional climate conditions by creating localized water availability that exceeds what would be predicted from climate alone.

Propagule pressure—the quantity and frequency of arriving individuals—is widely recognized as a critical determinant of invasion success (Lockwood et al. 2009; Simberloff 2009; Williams et al. 2021). High propagule pressure can overcome demographic stochasticity, Allee effects, and environmental barriers that might otherwise prevent establishment (Lockwood et al. 2005; Zhao et al. 2023). In heterogeneous environments, the interaction between propagule pressure and microhabitat quality may be particularly important, as sufficient propagule input might compensate for suboptimal conditions. Furthermore, the characteristics of the propagules themselves, such as their origin or the environmental conditions experienced by parent generations, can influence establishment success and population response to new environmental pressures (Warren et al. 2012; Vedder et al. 2021). Therefore, we hypothesize that moisture‐dependent plant species can achieve enhanced survival in novel arid environments through the synergistic effects of topographically created moisture refugia and sufficient propagule pressure.

To test this hypothesis, we use the invasion of giant ragweed ( *Ambrosia trifida* L.) as a model system. An annual invasive weed native to North America and typically adapted to moist environments (Montagnani et al. 2017) has caused severe ecological and agricultural damage in multiple regions worldwide (Page and Nurse 2015; Regnier et al. 2016; Schaffner et al. 2020). In recent years, this species has exhibited a perplexing pattern of rapid invasion and expansion in the Yili Valley, an arid region of Northwest China (Dong et al. 2020; Wang et al. 2022). This invasion into an arid environment appears to challenge expectations based on the species' known climatic preferences from its native and other invaded ranges. This phenomenon provides an ideal case study for investigating the ecological mechanisms that enable the invasion of a wetland plant in a seemingly unsuitable arid environment.

This study aims to: (1) quantify the climatic divergence between the Yili Valley and the typical range of *A. trifida* ; (2) experimentally assess how topographically driven moisture gradients influence seedling establishment and plant survival; and (3) evaluate the role of propagule pressure and its interaction with moisture availability in facilitating establishment, while also exploring potential differences among propagules from different collection years.

## 2 Materials and Methods

### 2.1 Study Area

**FIGURE 1:**
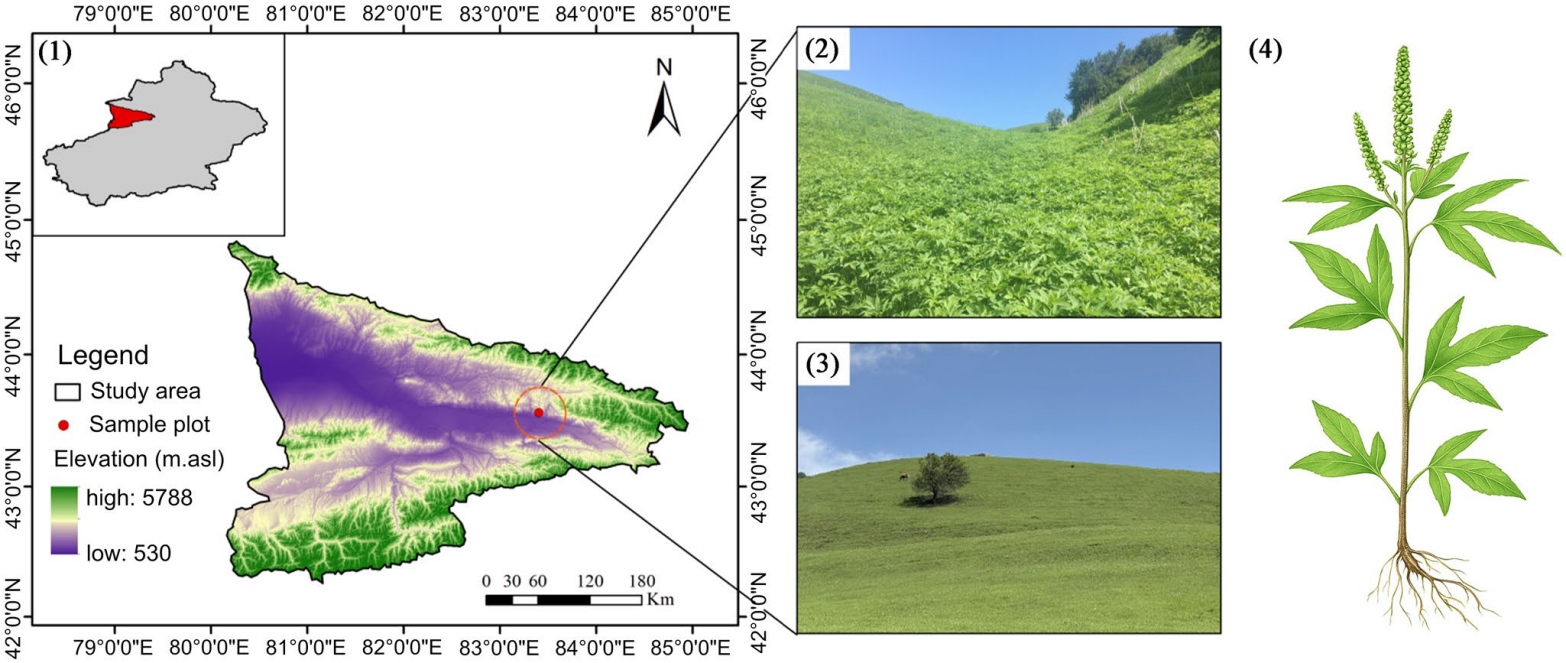
Overview of the study area. (1) Location of the Yili Valley, where *Ambrosia trifida* invasion occurs. (2) Grassland areas already invaded by *A. trifida* . (3) Uninvaded hilly grasslands. (4) Morphological diagram of *A. trifida* .

This study was conducted in the Yili Valley (80°09′42″84°56′50″E, 42°14′16″–44°53′30″N), located at the westernmost end of the Western Tianshan Mountains in Xinjiang, China (Figure 1). The region has a typical continental temperate arid climate, with a mean annual temperature of 10.4°C and mean annual precipitation of 417.6 mm. However, precipitation distribution is highly uneven, mainly concentrated in high‐altitude mountainous areas, while the annual precipitation in plain areas is only 200–350 mm. The mean annual evaporation ranges from 1250 to 1800 mm, exhibiting typical arid climate characteristics. *Ambrosia trifida* predominantly invades the hilly grasslands of this region, where the primary soil type is Kastanozem. The typical hilly topography of this region leads to microhabitat differentiation among slope positions, particularly forming distinct gradients in soil moisture conditions. This provides a natural experimental platform for studying how invasive plants experience selection and exhibit adaptive potential in heterogeneous environments (Zhang 2005; Dong et al. 2020; Yang et al. 2024).

### 2.2 Climatic Niche Analysis

To assess climatic niche differentiation between native and invaded populations of *A. trifida* , we employed a comprehensive analytical framework based on the ecospat R package (Feijó et al. 2021; Alhajeri and Fourcade 2019). This approach addresses the fundamental versus realized niche distinction by incorporating background climate availability analysis.

Data preparation: We compiled occurrence records from three categories: native range populations, Yili Valley populations, and background points randomly sampled from the native range to represent available climate space. All occurrence data were filtered to remove duplicates and records with coordinate uncertainty > 1 km. We extracted 19 bioclimatic variables (Bio1‐Bio19) from WorldClim 2.1 at 10 arc‐minutes resolution for each occurrence point and background location.

### 2.3 Experimental Design

#### 2.3.1 Seed Material and Viability Assessment

Seeds were collected from established *A. trifida* populations in the Yili Valley during October–November of 2010, 2013, 2016, 2019, and 2022. These seeds were stored in a seed storage cabinet (Model CZ‐1000FC). To account for potential viability differences due to storage duration, we conducted standardized germination tests. For each collection year, 6 replicates of 25 seeds were germinated in growth chambers (25/10°C day/night, 12 h photoperiod). Germination was recorded daily for 30 days.

To ensure comparable effective propagule pressure across treatments, we adjusted sowing densities based on observed germination rates:

(1) $$\mathrm{AD}=\mathrm{TD}\times \left(\mathrm{RGR}/\mathrm{TGR}\right)$$

where AD is the adjusted density (seeds m^−2^) representing the actual number of seeds sown per m^2^ for each collection year; TD is the target density (seeds m^−2^) representing the desired number of viable seeds per m^2^ across all treatments; RGR is the reference germination rate (proportion, 0–1) determined from the germination test of the 2022 seed collection; and TGR is the treatment germination rate (proportion, 0–1) determined from the germination test of each respective collection year (2010, 2013, 2016, 2019, 2022). The 2022 collection served as the reference as it represented the most recent collection.

#### 2.3.2 Field Experiment

This study examined how topography and propagule pressure affect *A. trifida* 's establishment in arid areas and its adaptation mechanisms. It was conducted in Yili Valley's hilly grasslands, which are invaded but not fully occupied by *A. trifida* . The experiment tested the effects of ecological factors and looked for adaptive signals, simulating different propagule pressures through manual sowing.

*Ambrosia trifida* seeds collected from Yili Valley in 2010, 2013, 2016, 2019, and 2022 were used to establish propagule pressure gradients while exploring potential adaptive changes during invasion progression.

##### 2.3.2.1 Field Experiment Design

In November 2023, a propagule pressure experiment was conducted at three east–west oriented grassland sites in Yili Valley. Each site included three habitats (upper, middle, lower hillside) representing moisture gradients, with three randomized blocks per habitat. Twenty‐five treatment combinations (5 seed collection years × 5 sowing densities: 5, 10, 20, 50, 100 seeds/m^2^) were randomly assigned to 1 m × 1 m subplots within each block. Seed numbers were adjusted to achieve equivalent viable seed densities across collection years, with specific sowing numbers for each treatment calculated as described in the previous section. The experiment comprised 675 subplots total (3 sites × 3 habitats × 3 blocks × 25 treatments).

Environmental Monitoring and Data Collection: Soil moisture sensors (WatchDog 1400) were installed at a depth of 15 cm in each habitat (*n* = 9 total), recording hourly for 1 year. Soil samples were collected along 18 m transects (10 samples per habitat at 2 m intervals), pooled by habitat, and analyzed for organic matter, available/total N‐P‐K, and pH. Seedling establishment was surveyed in early May 2024 during peak germination, with the establishment rate calculated as: (established individuals)/(viable seeds sown). A supplementary count occurred in late July during flowering. All aboveground biomass was harvested in early October. Subsequent *A. trifida* plants will be removed over 2 years to prevent anthropogenic spread.

### 2.4 Analytical Methods

Data analysis and visualization were performed using R (version 4.4.2) with the ‘ecospat’ package for niche analysis, and the ‘lme4’ and ‘lmerTest’ packages for mixed‐effects models, and SPSS (version 2019) or Origin (version 2021b) for visualizations.

#### 2.4.1 Climatic Niche Analysis

For the climatic niche analysis, we first performed Principal Component Analysis (PCA) on standardized bioclimatic variables using all occurrence and background points to define the global environmental space (Zomer et al. 2022), retaining the first two principal components based on their cumulative explained variance. We then applied the ecospat gridding procedure to quantify climatic niches in this two‐dimensional PCA space, creating density grids for each population (native and Yili) using kernel density estimation with a resolution of 100 × 100 cells, where the background climate space was used to weight the available environment (Escobar et al. 2016).

Niche overlap was quantified using Schoener's *D* index (ranging from 0 for no overlap to 1 for complete overlap), and we performed two statistical tests using 1000 randomizations: (1) niche equivalency test to assess whether niches are statistically indistinguishable, and (2) niche similarity test to determine whether observed overlap exceeds random expectations under background climate constraints. We further quantified three niche dynamics components: stability (proportion of native niche retained in the invaded range), expansion (proportion of invaded niche representing novel climatic conditions), and unfilling (proportion of native niche unoccupied in the invaded range).

Finally, to assess whether Yili Valley climatic conditions exist within the native range, we calculated the proportion of background points falling within the climatic space occupied by Yili populations, expanded by 10% to account for environmental variation (Mungi et al. 2020).

#### 2.4.2 Analysis of Topographic and Propagule Pressure Effects on *A. trifida* Establishment and Survival

To assess the effects of slope position (a proxy for moisture), sowing density (a proxy for propagule pressure), and seed collection year on the establishment and survival of *A. trifida* , we fitted linear mixed‐effects models (LMMs). The response variables were the arcsine‐square root transformed establishment rate (seedling number/viable seed number) and final survival rate (mature plant number/viable seed number) to meet model assumptions.

In the LMMs, slope position, sowing density, and seed collection year, along with all their two‐way and three‐way interactions, were included as fixed effects. ‘Site’ and ‘Block’ (nested within ‘Site’) were included as random effects to account for the spatial structure of the experiment. We checked for multicollinearity among fixed effects using the Variance Inflation Factor (VIF), and all VIF values were below 5, indicating no significant collinearity issues. Model selection was performed using a backward elimination approach based on the Akaike Information Criterion (AIC).

We started with the full model and sequentially removed nonsignificant interaction terms to arrive at the most parsimonious model. The significance of fixed effects was assessed using F‐tests with Satterthwaite's degrees of freedom approximation. Model assumptions were validated by visually inspecting plots of residuals versus fitted values and quantile‐quantile plots of the residuals to check for homogeneity of variance and normality (Portet 2020).

#### 2.4.3 Seed Viability and Environmental Factor Analyses

Seed viability differences among collection years and soil chemical properties and moisture content across different slope positions were tested using one‐way ANOVA followed by Tukey's HSD post hoc test.

## 3 Results

### 3.1 Niche Differentiation Between Native and Invaded Ranges

**FIGURE 2:**
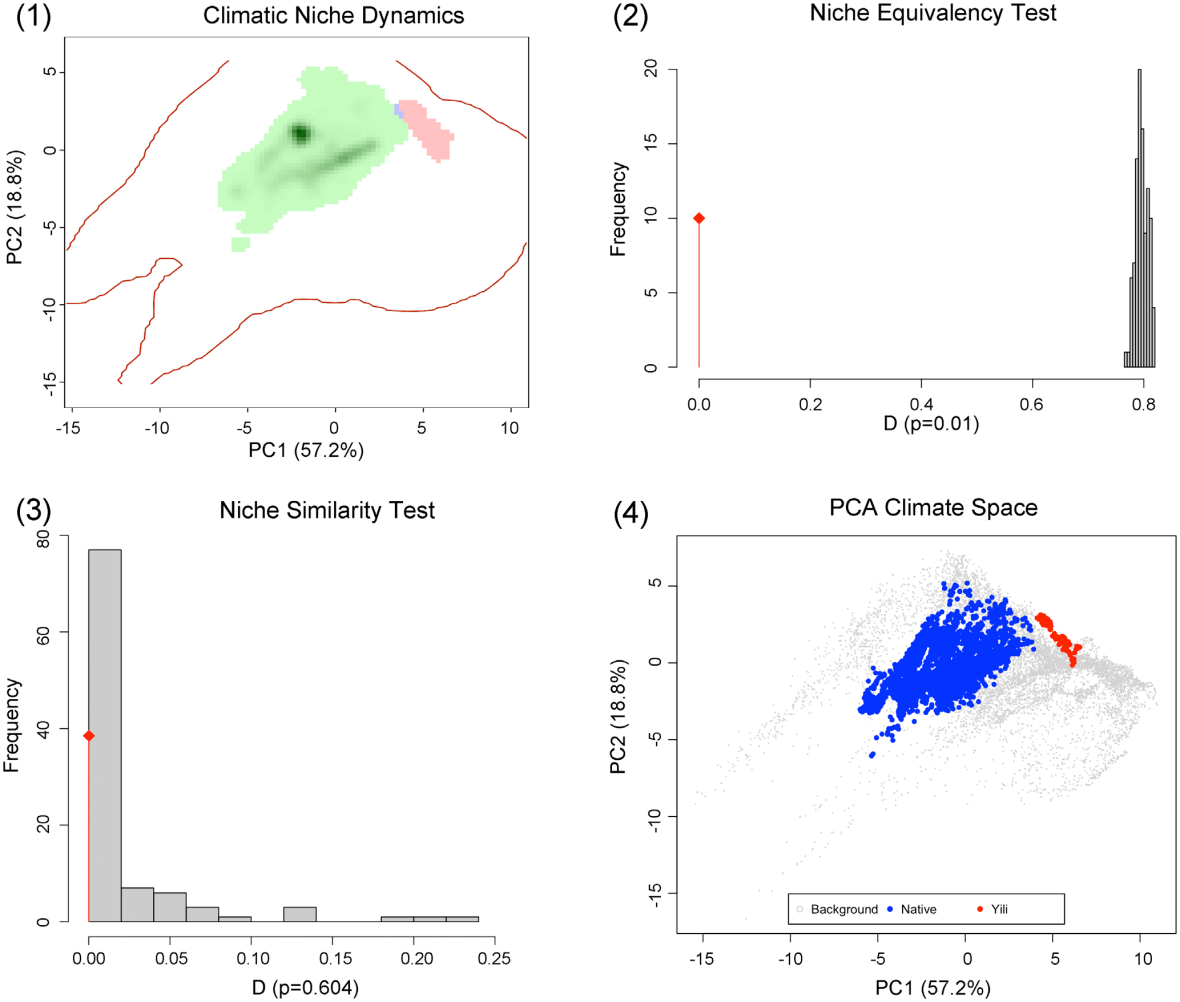
Climatic niche analysis of *Ambrosia trifida* between native range and Yili Valley populations. (1) Climatic niche dynamics showing kernel density distributions in PCA space, with green shading representing native range niche density and red shading representing Yili Valley niche density. Red contour lines delineate the 25% quantile boundaries of each niche. (2) Niche equivalency test histogram showing the distribution of Schoener's D values from 1000 randomizations (gray bars), with the observed D value indicated by the red diamond (*p* = 0.01). (3) Niche similarity test histogram showing the distribution of Schoener's D values from 1000 randomizations (gray bars), with the observed D value indicated by the red diamond (*p* = 0.604). (4) PCA climate space showing the distribution of background points from the native range (gray dots), native range occurrences (blue dots), and Yili Valley occurrences (red dots) along the first two principal components, which explain 57.2% and 18.8% of climatic variance, respectively.

The Principal Component Analysis of 19 bioclimatic variables explained 76.0% of the total climatic variance in the first two axes (PC1: 57.2%, PC2: 18.8%) (Figure 2, (1) and (4)). Native and Yili Valley populations occupied distinct regions of the PCA climate space with minimal spatial overlap.

**FIGURE 3:**
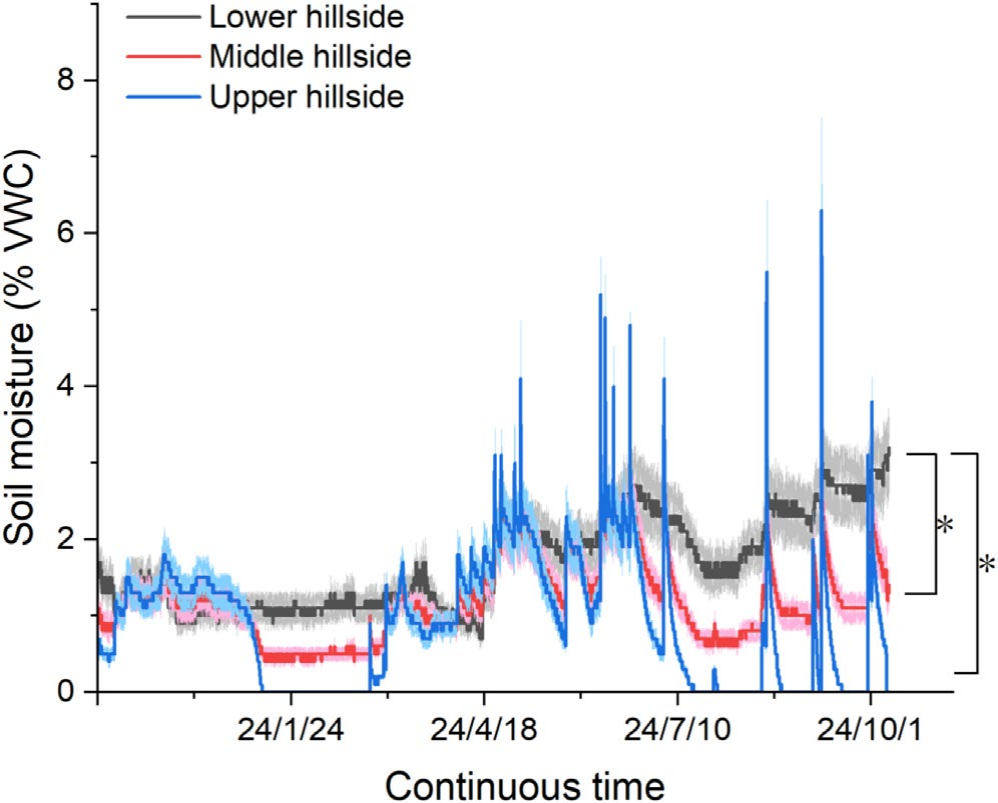
Variation in soil moisture at different slope positions over a year. Soil moisture content is significantly higher at the lower hillside compared to the middle and upper hillside. “*” indicates a significant difference between the two groups at *p* < 0.05 level.

Niche overlap analysis yielded a Schoener's D value of 0.000027 and Hellinger's *I* value of 0.0005. The niche equivalency test produced a *p* value of 0.01, while the niche similarity test yielded a *p*‐value of 0.604 (Figure 2, (2) and (3)). Niche dynamics analysis showed stability of 0.014 (1.4%), expansion of 0.986 (98.6%), and unfilling of 1.000 (100%). Background climate availability analysis revealed that 13.1% of randomly sampled points within the native range exhibited climatic conditions similar to those in the Yili Valley.

### 3.2 Microhabitat Moisture Gradients

Soil water content exhibited significant and persistent differences among slope positions throughout the growing season. The volumetric soil water content at the lower hillside position generally remained between 2% and 3% during the growing season, significantly higher than that at the middle and upper hillside positions, thus forming a distinct moisture gradient (Figure 3, Table 1). However, there were no significant differences in major soil nutrient content (including organic matter, available nitrogen, available phosphorus, available potassium, total nitrogen, total phosphorus, total potassium, and pH) among the different slope positions (Table 1). This indicates that moisture availability is the primary driver of microenvironmental heterogeneity among slope positions in this study area, creating varying intensities of selection pressure on the invasive plant.

**TABLE 1:** Soil properties at different slope positions.

Soil properties	Lower hillside	Middle hillside	Upper hillside
Organic matter (g/kg)	26.11 ± 1.01	26.45 ± 1.65	25.83 ± 2.16
Available N (mg/kg)	41.22 ± 1.55	40.89 ± 1.60	39.67 ± 3.07
Available P (mg/kg)	3.16 ± 0.28	3.22 ± 0.30	3.06 ± 0.38
Available K (mg/kg)	155.78 ± 3.51	152.10 ± 3.60	157.00 ± 5.15
Total N (g/kg)	1.72 ± 0.04	1.70 ± 0.02	1.68 ± 0.05
Total P (g/kg)	1.01 ± 0.02	1.02 ± 0.03	0.99 ± 0.04
Total K (g/kg)	23.67 ± 2.34	24.10 ± 3.40	22.37 ± 4.11
pH	7.88 ± 0.27	7.85 ± 0.31	7.92 ± 0.26
Volumetric water content	1.94 ± 0.270.01 a	1.43 ± 0.01 b	1.15 ± 0.02 c

*Note:* Differences in soil nutrient contents across different slope positions were not significant, while significant differences were observed in soil volumetric water content. Different letters indicate significant differences at the *p* < 0.05 level.

### 3.3 Effects on Establishment and Survival

#### 3.3.1 Differences in Seed Viability Across Storage Years and Calibration

**FIGURE 4:**
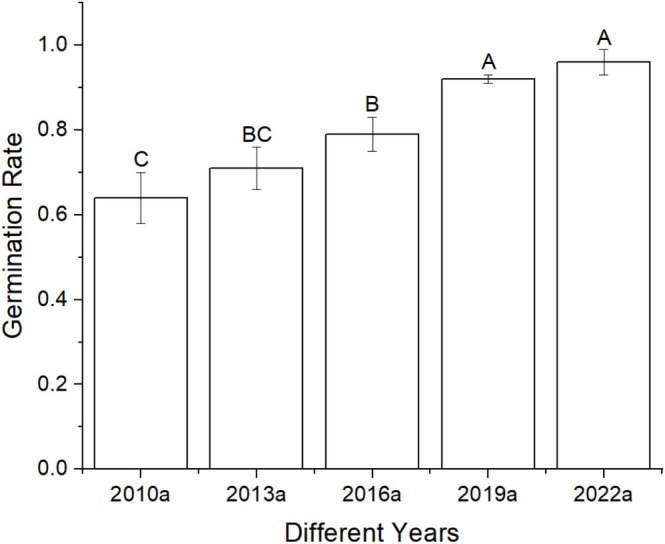
Differences in seed germination rates across various storage durations. Labels 2010a–2022a denote seeds stored for 1–13 years, respectively. Different letters indicate significant differences.

Significant differences in seed vigor were observed among *A. trifida* seeds stored for varying durations (Figure 4). Seeds stored for 1 year (2022a) demonstrated a significantly higher germination rate compared to seeds stored for 7 years or more. To ensure that treatments within the propagule pressure simulation experiments had comparable germination rates, thereby standardizing the germination rates of seeds stored for different durations, the germination rate of the most recently stored seeds (2022a) was used as a baseline. Other years' seed germination rates were calibrated accordingly, resulting in the actual seed numbers required for each treatment during field sowing (Table S1).

#### 3.3.2 Statistical Analysis of Establishment and Survival Factors

Linear mixed‐effects model analysis revealed distinct patterns of factor importance between seedling establishment and plant survival stages (Table 2). For seedling establishment, slope position (*F* = 109.77, *p* < 0.001) and sowing density (*F* = 225.55, *p* < 0.001) were the only significant predictors retained (*R* ^2^ = 0.638). Establishment rates decreased significantly on middle (*β* = −0.146, SE = 0.012) and upper slopes (*β* = −0.151, SE = 0.012) relative to lower slopes, while density showed a strong positive linear effect (*β* = 0.303, SE = 0.018). A significant slope × density interaction (*F* = 5.18, *p* < 0.001) indicated varying density responses across slope positions.

**TABLE 2:** Linear mixed‐effects model results for seedling establishment and plant survival rates.

Effect/Parameter	Seedling establishment rate		Plant survival rate	
	*F* value	*p*	*F*	*p*
*ANOVA results*				
Slope position	109.77	< 0.001***	53.67	< 0.001***
Sowing density	225.55	< 0.001***	185.43	< 0.001***
Seed storage years	—	—	20.24	< 0.001***
Slope × Density	5.18	< 0.001***	6.25	< 0.001***
Density × Year	—	—	2.30	0.003**
	*β* (SE)	*p*	*β* (SE)	*p*
Model coefficients				
Intercept (Lower slope)	0.398 (0.008)	< 0.001***	0.124 (0.008)	< 0.001***
Slope position				
Middle versus Lower	−0.146 (0.012)	< 0.001***	−0.038 (0.007)	< 0.001***
Upper versus Lower	−0.151 (0.012)	< 0.001***	−0.075 (0.005)	< 0.001***
Sowing density				
Linear trend	0.303 (0.018)	< 0.001***	0.222 (0.017)	< 0.001***
Seed storage years				
Year 13a	—	—	−0.035 (0.009)	< 0.001***
Year 1a	—	—	0.038 (0.007)	< 0.001***
Year 4a	—	—	0.036 (0.009)	< 0.001***
Key interactions				
Upper slope × Density.L	0.054 (0.026)	0.036*	−0.111 (0.016)	< 0.001***
Upper slope × Density.C	−0.111 (0.026)	< 0.001***	—	—
Upper slope × Density.Q	0.096 (0.026)	< 0.001***	—	—
Middle slope × Density.L	—	—	−0.054 (0.015)	< 0.001***
Density.L × Year13a	—	—	−0.056 (0.021)	0.008**
Density.L × Year1a	—	—	0.049 (0.022)	0.019*
Model summary				
*R* ^2^	0.638		0.614	
Adjusted *R* ^2^	0.630		0.593	
Residual SE	0.123		0.077	

*Note:* Results of linear mixed‐effects models examining the effects of slope position (proxy for soil moisture: lower, middle, upper slopes), sowing density (propagule pressure: 5–100 viable seeds/m^2^), and seed storage duration (1–13 years) on arcsine‐square root transformed establishment and survival rates. ANOVA results show overall significance tests using Type III *F*‐tests; Model coefficients present parameter estimates (*β*) with standard errors (SE) indicating effect direction and magnitude; Key interactions show only significant terms retained after backward elimination based on AIC. Models included Site and Block (nested) as random effects. The symbol “—” indicates terms not retained in final models. Sowing density treated as ordered factor with orthogonal polynomial contrasts: L (linear trend), Q (quadratic trend), C (cubic trend) representing different patterns of density response. *R* ^2^ indicates proportion of variance explained.

*** *p* < 0.001.

** *p* < 0.01.

* *p* < 0.05.

For plant survival, slope position (*F* = 53.67, *p* < 0.001), sowing density (*F* = 185.43, *p* < 0.001), and seed storage years (*F* = 20.24, *p* < 0.001) were all significant (*R* ^2^ = 0.614). Effect patterns were similar to establishment but with smaller magnitudes (middle slope: *β* = −0.038; upper slope: *β* = −0.075; density: *β* = 0.222). Seeds stored for 13 years showed reduced survival (*β* = −0.035, SE = 0.009), with a significant density × year interaction (*F* = 2.30, *p* = 0.003).

**FIGURE 5:**
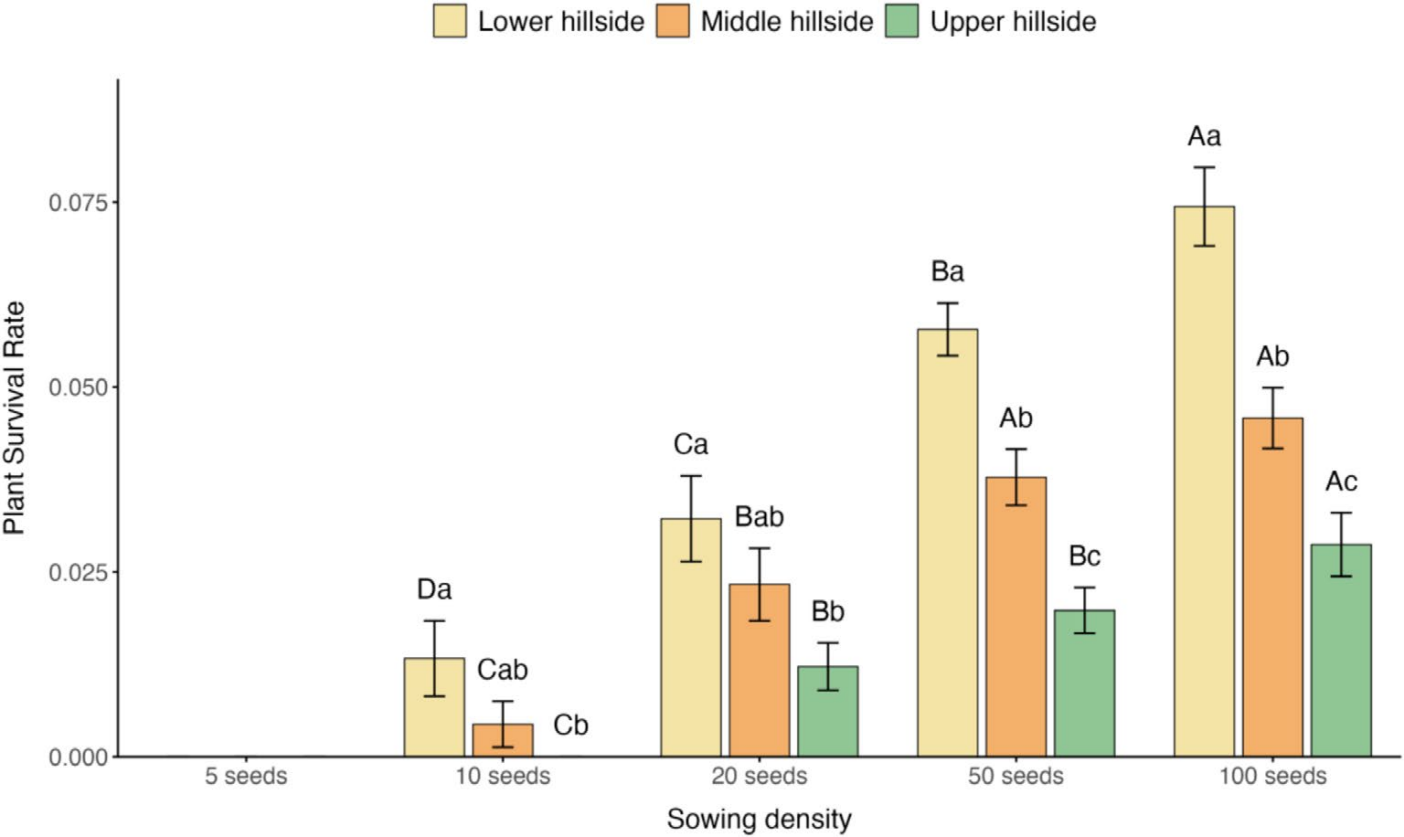
Final survival rates of *Ambrosia trifida* plants at different slope positions under varying seeding densities. Different uppercase letters indicate significant differences among treatments within the same slope position, while different lowercase letters indicate significant differences among slope positions for the same treatment.

The slope × density interaction was evident throughout both establishment and survival stages, with lower slopes showing the strongest positive responses to increased propagule pressure, while middle and upper slopes showed progressively weaker but still significant positive responses (Figure 5).

## 4 Discussion

This study reveals key ecological mechanisms by which topography‐driven microenvironmental heterogeneity and propagule pressure enable the wetland plant *A. trifida* to successfully invade the arid Yili Valley. Results demonstrated enhanced moisture stress tolerance, with establishment occurring at precipitation levels (200 mm) far below typical species requirements (> 800 mm). Microhabitat refugia and compensatory propagule pressure facilitated population persistence in climatically marginal environments. Temporal variation in seed performance across collection years suggests increasing tolerance during invasion progression, potentially indicating adaptive processes. These findings challenge traditional climate‐matching invasion risk assessments and highlight the importance of fine‐scale ecological mechanisms in facilitating biological invasions under novel environmental conditions.

### 4.1 Climatic Niche Expansion and the Role of Microenvironmental Refugia

Our climatic niche analysis demonstrates that *A. trifida* invasion in the Yili Valley represents genuine niche expansion, with 98.6% of occupied climatic space constituting novel conditions relative to the native range (Schoener's *D* = 0.000027). This challenges the traditional paradigm that species distributions are primarily constrained by broad climatic tolerances (Liu et al. 2020; Guerra‐Coss et al. 2021; Ángel‐Vallejo et al. 2024). Despite mean annual precipitation (200 mm) being far below typical requirements for this wetland species (> 800 mm), successful establishment occurred consistently across experimental sites (Xia et al. 2021).

The resolution to this apparent paradox lies in topographically driven microenvironmental heterogeneity (Puerta‐Pinero et al. 2007; Nemes‐Kókai et al. 2024; Chardon et al. 2024). Lower slope positions maintain significantly higher soil moisture than middle and upper slopes throughout the growing season, while soil nutrients show no significant variation among positions. This moisture accumulation likely results from runoff concentration and reduced evaporation in topographic depressions, creating critical refugia within an otherwise unsuitable climate (Moore et al. 1988; Le and Kumar 2014).

These findings align with emerging evidence that microhabitat heterogeneity facilitates species persistence in climatically marginal areas (Pahl et al. 2013; Nelson et al. 2021; Weston et al. 2021). Such refugia may serve as crucial “stepping stones” enabling initial establishment in otherwise unsuitable regions, suggesting that correlative distribution models based on regional climate averages may systematically underestimate invasion potential in topographically complex landscapes.

### 4.2 Propagule Pressure as a Compensatory Mechanism and the Influence of Seed Source Variation

Propagule pressure emerged as the strongest predictor of establishment success (*F* = 225.55, *p* < 0.001), with the significant slope × density interaction revealing that high propagule input can partially compensate for suboptimal moisture conditions. This compensatory effect was most pronounced at water‐limited middle and upper slopes, supporting theoretical predictions that propagule pressure overcomes environmental barriers through demographic rescue and increased microsite sampling probability (Holle and Simberloff 2005; Gertzen et al. 2011).

Notably, seed collection year significantly affected long‐term survival (*F* = 20.24, *p* < 0.001) but not initial establishment, with 2010‐collected seeds showing reduced performance despite germination rate standardization. While direct evidence for evolutionary adaptation remains limited, this temporal variation suggests potential population‐level changes during invasion progression (Orbán et al. 2021; Mircea et al. 2023). Similar patterns have been attributed to rapid adaptation in other invasive systems, though alternative explanations including maternal effects and transgenerational environmental influences cannot be excluded (Mousseau and Fox 1998; Wolf and Wade 2009).

The density × year interaction indicates that propagule pressure effects vary with seed source characteristics, highlighting the importance of considering propagule quality alongside quantity in invasion assessments (Colautti et al. 2006; Hufbauer et al. 2013; Alzate et al. 2020). From a management perspective, these results suggest that preventing initial seed input may be particularly effective in climatically marginal regions, where environmental limitations already constrain establishment, and early intervention may target less locally‐adapted propagules (Walck et al. 2011).

## 5 Conclusions

This study investigated how giant ragweed, a wetland invasive plant, successfully establishes in the arid Yili Valley despite climatically unsuitable regional conditions. Our integrated approach combining climatic niche analysis and field experiments revealed three key mechanisms underlying this invasion success. First, *A. trifida* achieved 98.6% climatic niche expansion by exploiting topographically driven moisture refugia, where lower slope positions provide critical water accumulation within an otherwise hostile environment. Second, propagule pressure serves as a compensatory mechanism, with the significant slope × density interaction demonstrating that high seed input can partially overcome moisture limitations. Third, temporal variation in seed source performance suggests potential population‐level changes during invasion progression, though direct evolutionary evidence remains limited.

These findings contribute significantly to invasion ecology theory by challenging climate‐matching paradigms and demonstrating that microenvironmental heterogeneity can facilitate establishment in climatically marginal regions. Practically, our results inform management strategies by identifying high‐risk microsites for early detection efforts and emphasizing the effectiveness of preventing initial propagule input in environmentally constrained landscapes. Future research should employ common garden experiments and genomic approaches to definitively assess adaptive evolution during invasion, while extending monitoring periods to capture long‐term population dynamics.

## Author Contributions

**Shengtianzi Dong:** conceptualization (equal), data curation (equal), investigation (equal), writing – original draft (equal). **Wenxuan Zhao:** data curation (supporting), investigation (supporting). **Tiantian Qin:** investigation (supporting), software (supporting). **Hongyang Chen:** investigation (supporting), software (supporting). **Wenchao Guo:** investigation (supporting), visualization (supporting). **Hanyue Wang:** conceptualization (lead), funding acquisition (lead), resources (lead), writing – review and editing (lead). **Hegan Dong:** resources (supporting), writing – review and editing (lead).

## Conflicts of Interest

The authors declare no conflicts of interest.

## Supporting information


**Table S1:** Source data for the manuscript.

## Data Availability

Data available of this article can be found online at https://doi.org/10.6084/m9.figshare.27988220.v1.
